# Electroconvulsive Therapy for Treatment-Resistant Late-Onset Bipolar Disorder

**DOI:** 10.7759/cureus.89997

**Published:** 2025-08-13

**Authors:** Sarah Garikana, Mohamed Elrafei

**Affiliations:** 1 Psychiatry, Icahn School of Medicine at Mount Sinai, New York City, USA; 2 Psychiatry, State University of New York Upstate Medical University, Syracuse, USA; 3 Psychiatry, Wayne Behavioral Service, Wayne, USA

**Keywords:** bipolar disorder (bd), electroconvulsive therapy (ect), late-onset bipolar disorder, occupational stress, treatment-resistant psychiatric disorders

## Abstract

This paper presents the case of a woman with a psychiatric history of major depressive disorder and generalized anxiety disorder, who experienced her first manic episode at age 50, likely triggered by occupational stress. She was diagnosed with bipolar I disorder and underwent multiple trials of antipsychotic and mood-stabilizing medications, including cariprazine, olanzapine, lumateperone, lithium, and valproate. Despite several pharmacological interventions, the patient continued to exhibit mood instability, frequent panic attacks, and functional decline. Following a course of 12 electroconvulsive therapy (ECT) sessions, she showed significant clinical improvement, including remission of manic symptoms, reduction in panic attacks, and regained occupational functioning.

## Introduction

Bipolar disorder is a chronic psychiatric illness characterized by fluctuations in mood state and energy that include emotional highs (mania or hypomania) and lows (depression) [1,2]. Bipolar disorder is typically classified into three main subtypes: bipolar I disorder, involving at least one episode of full-blown mania or mixed episode (manic and depressive symptoms); bipolar II disorder, characterized by several protracted depressive episodes and at least one hypomanic episode, but no manic episodes; and cyclothymic disorder, which includes periods of hypomanic and depressive symptoms that do not meet meet the criteria for full-blown hypomanic or major depressive episodes [3]. The age at onset (AAO) is a critical factor that impacts the course and treatment outcomes of the disorder. Several studies describe the AAO of bipolar disorder as a multimodal distribution, with both bimodal (early and late onset) and trimodal (early, intermediate, and late onset) patterns reported [4,5]. The International Society for Bipolar Disorders Task Force recommends classifying bipolar disorder into late-onset bipolar disorder (LOBD) if onset at or after age 50, else as early-onset bipolar disorder (EOBD) [6].

LOBD accounts for approximately 5-10% of all bipolar cases [7,8]. These patients often develop treatment resistance and have higher mortality rates when compared to EOBD patients [9]. It is also frequently associated with neurological diseases, cognitive decline, or other somatic issues [10]. Despite these clinical concerns, there is limited evidence to guide pharmacologic or non-pharmacologic treatment approaches for LOBD. Most of the practice relies on extrapolations from recommendations in younger populations or general adult samples, which may not account for age-related vulnerabilities [10].

Electroconvulsive therapy (ECT) is a widely used medical treatment for severe mood and psychotic disorders [11]. ECT involves a brief electrical stimulation of the brain while the patient is under anesthesia, inducing a generalized seizure that typically lasts for 20-60 seconds. A standard course consists of 6-12 treatments spaced over a period of two to four weeks [11]. ECT is a highly effective intervention for several mood disorders, including bipolar depression and mania, with strong evidence supporting its safety and efficacy [12,13]. Though the exact mechanism of action for ECT is unclear, recent research suggests that it disrupts neural circuits and enhances neuroplasticity, enabling the brain to rewire to more optimal patterns [14]. Despite its effectiveness, systematic data on the use of ECT in LOBD patients are lacking. Existing evidence comprises a few case reports [15,16] and case series [17], rather than large-scale randomized controlled trials. Nonetheless, most of the available literature suggests that ECT is a safe and effective treatment in older adults, particularly in cases of treatment-resistant bipolar disorder [18].

This paper presents the case of a woman with a history of recurrent major depressive disorder and generalized anxiety who developed her first manic episode at age 50, likely triggered by occupational stress. She was diagnosed with bipolar I disorder per Diagnostic and Statistical Manual of Mental Disorders, 5th Edition (DSM-5) criteria [19]. Despite multiple antipsychotic trials over six months, her symptoms continued to worsen, resulting in social and occupational dysfunction and eventually hospitalization. Following a course of ECT, she demonstrated significant clinical improvement. This case highlights the role of ECT as an important treatment option for treatment-resistant bipolar disorder [20], particularly in late-onset cases.

## Case presentation

The patient is a 50-year-old married woman with one child. She was employed in human resources, tasked with facilitating employee terminations, a responsibility she described as a major source of occupational stress. She came to the clinic voluntarily for an in-person visit, accompanied by her husband. At the time of assessment, she was exhibiting symptoms consistent with a full-blown manic episode. Her mood was irritable, and she reported several days of severely reduced sleep, heightened distractibility, elevated energy, and extreme mood fluctuations ranging from euphoria to intense irritability. Her husband observed that she had become excessively talkative and was using profane language, which was very uncharacteristic for her. The manic episode had persisted for over a week by the time of presentation. She denied any obsessive, intrusive, and persistent thoughts or compulsive/ritualistic acts. She also denied hallucinations, delusions, or substance use. A comprehensive medical evaluation was conducted, including primary care consultation, physical and neurological examinations, and laboratory work, to rule out any underlying organic causes.

The patient's mother had a history of alcohol use disorder, but there were no other significant familial psychiatric conditions. The patient herself had a longstanding history of mental illness. At age 33, she experienced postpartum depression with suicidal ideation following childbirth. Three years later, she was diagnosed with major depressive disorder (recurrent, severe without psychotic features) and generalized anxiety disorder. She was first started on citalopram 5 mg daily, and over the years, the dosage was increased to manage her symptoms. During this time, she remained free of major depressive, anxious, or manic episodes. At the time of her presentation, she was being treated with citalopram 40 mg daily and alprazolam 0.5 mg daily, and was actively participating in group psychotherapy. Her medical history was also notable for a diagnosis of type 2 diabetes mellitus, made seven years prior at age 43. She was receiving metformin 500 mg, losartan 25 mg, and fenofibrate 54 mg daily for the management of her diabetes. Additionally, she had a long-standing history of obesity. At the time of her visit, her weight was 220 pounds, height 5 feet 11 inches, a BMI of 30, and a hemoglobin A1C level of 7%. All her medications at the time of her visit are listed in Table 1.

**Table 1:** Medications at the time of her presentation.

Medication	Daily dosage
Citalopram	40 mg
Alprazolam	0.5 mg
Metformin	500 mg
Losartan	25 mg
Fenofibrate	54 mg

Based on clinical assessment and in accordance with DSM-5 criteria [13], she was diagnosed with bipolar I disorder. Pharmacological treatment was initiated with cariprazine 3 mg daily, and topiramate 25 mg was added to assist with mood stabilization. Her existing medications remained unchanged. Despite treatment, the patient showed minimal clinical improvement over the following four months. The following symptoms continued to persist: depressed mood, social withdrawal, and difficulty with focus and concentration. She experienced frequent, untriggered panic attacks throughout the day and continued to suffer from chronic insomnia. Behavioral observations included constant pacing and difficulty remaining seated during group therapy sessions.

Given the lack of progress, she was subsequently trialed on multiple antipsychotic medications without any significant improvement. She was initially maintained on cariprazine 3 mg daily for nearly two months, followed by a trial of olanzapine, beginning at 10 mg daily for two weeks and then increased to 15 mg daily over the next six weeks. This was followed by a one-month trial of lumateperone at 42 mg daily. Finally, she was transitioned to quetiapine, starting at 300 mg daily for one week and then increased to 400 mg for another three weeks. However, these interventions failed to yield significant improvement. As a result, she went on short-term disability leave from work and enrolled in a partial hospitalization program for structured therapy. She also later required inpatient psychiatric hospitalization due to the development of akathisia.

Given the limited response to pharmacotherapy, the patient was recommended to pursue ECT. After a detailed consultation with a psychiatrist specialized in ECT, the patient and her family agreed to proceed. Over the course of the next two months, the patient underwent 12 ECT sessions. For each session, sedation was induced using propofol, followed by the administration of succinylcholine to prevent prolonged convulsions. Electrodes were placed bitemporally, and brief pulse stimulation was delivered. Following the ECT course, the patient demonstrated substantial clinical improvement. Her panic attacks significantly decreased in both frequency and severity. She experienced improvements in mood, focus, and concentration. She began to re-engage socially and was ultimately able to return to full-time employment. As part of her ongoing maintenance regimen, she was prescribed the medications listed in Table 2.

**Table 2:** Medications prescribed for the ongoing maintenance regimen.

Medication	Daily dosage
Lithium	50 mg
Quetiapine	300 mg
Escitalopram	40 mg
Zolpidem	12.5 mg
Lorazepam	4 mg

## Discussion

LOBD is increasingly recognized as a clinically distinct subtype with unique treatment challenges [18]. Patients with late onset, like the subject in this case, often exhibit a treatment-resistant course of illness [10]. Her case fits the criteria for treatment-resistant bipolar disorder, which is usually defined as when a patient does not attain any sustained symptomatic relief following two adequate trials of pharmacologic treatments [20]. Our patient failed to respond to multiple trials of antipsychotic and mood-stabilizing medications and continued to exhibit significant mood instability, panic attacks, occupational and social decline, exemplifying the treatment-resistant trajectory.

Though underutilized in this population [17], ECT was critical in achieving symptom remission for our patient. This aligns with emerging but limited evidence supporting ECT’s efficacy in treatment-resistant bipolar disorder, particularly among older adults. Given the lack of randomized controlled trials focused specifically on LOBD, this case adds to the growing body of evidence advocating for ECT as a viable and effective intervention in such scenarios, as it offers a safe and effective treatment option for older adults [15,16,21].

Another interesting clinical aspect of this case is the patient’s history of type 2 diabetes mellitus. Bipolar disorder and diabetes often co-occur, and research suggests that there may be a pathophysiology linking the two disorders [22], with underlying autoimmune components. Additionally, diabetes and obesity, as observed in this case, can contribute to a treatment-resistant course of bipolar disorder [23]. Metabolic disorders can worsen the course of bipolar illness by increasing systemic inflammation, which may impair mood regulation [24]. Diabetes can also complicate pharmacological management of patients with mental health disorders [25]. This case emphasizes the need for integrated medical and psychiatric care in managing patients with such complex profiles.

## Conclusions

This case presents some of the challenges associated with LOBD, particularly when compounded by treatment resistance, medical comorbidities, and occupational stressors. The case demonstrates some of the limitations of conventional pharmacologic approaches in LOBD and highlights ECT as a safe and effective intervention in treatment-resistant cases. As research on LOBD remains limited, this case contributes to the growing evidence base supporting the role of ECT. However, the findings should be interpreted with caution. The specific nature of this case underscores the need for studies with larger sample sizes and rigorous methodological designs to validate the efficacy of ECT.
